# Abnormal CD13/HLA-DR Expression Pattern on Myeloblasts Predicts Development of Myeloid Neoplasia in Patients With Clonal Cytopenia of Undetermined Significance

**DOI:** 10.1093/ajcp/aqac083

**Published:** 2022-08-08

**Authors:** Dragan Jevremovic, Ahmad Nanaa, Susan M Geyer, Michael Timm, Haya Azouz, Cynthia Hengel, Alexander Reberg, Rong He, David Viswanatha, Mohamad E Salama, Min Shi, Horatiu Olteanu, Pedro Horna, Gregory Otteson, Patricia T Greipp, Zhuoer Xie, Hassan B Alkhateeb, William Hogan, Mark Litzow, Mrinal M Patnaik, Mithun Shah, Aref Al-Kali, Phuong L Nguyen

**Affiliations:** Division of Hematopathology, Mayo Clinic, Rochester, MN, USA; Division of Hematology, Mayo Clinic, Rochester, MN, USA; Department of Quantitative Health Sciences, Mayo Clinic, Rochester, MN, USA; Division of Hematopathology, Mayo Clinic, Rochester, MN, USA; Division of Hematology, Mayo Clinic, Rochester, MN, USA; Division of Hematopathology, Mayo Clinic, Rochester, MN, USA; Division of Hematopathology, Mayo Clinic, Rochester, MN, USA; Division of Hematopathology, Mayo Clinic, Rochester, MN, USA; Division of Hematopathology, Mayo Clinic, Rochester, MN, USA; Sonic Healthcare USA, Austin, TX, USA; Division of Hematopathology, Mayo Clinic, Rochester, MN, USA; Division of Hematopathology, Mayo Clinic, Rochester, MN, USA; Division of Hematopathology, Mayo Clinic, Rochester, MN, USA; Division of Hematopathology, Mayo Clinic, Rochester, MN, USA; Division of Hematopathology, Mayo Clinic, Rochester, MN, USA; Division of Laboratory Genetics and Genomics, Mayo Clinic, Rochester, MN, USA; Division of Hematology, Mayo Clinic, Rochester, MN, USA; Division of Hematology, Mayo Clinic, Rochester, MN, USA; Division of Hematology, Mayo Clinic, Rochester, MN, USA; Division of Hematology, Mayo Clinic, Rochester, MN, USA; Division of Hematology, Mayo Clinic, Rochester, MN, USA; Division of Hematology, Mayo Clinic, Rochester, MN, USA; Division of Hematology, Mayo Clinic, Rochester, MN, USA; Division of Hematopathology, Mayo Clinic, Rochester, MN, USA

**Keywords:** Flow cytometry, CCUS, MDS, Myeloid neoplasia

## Abstract

**Objectives:**

Patients with clonal cytopenia of undetermined significance (CCUS) are at increased risk of developing myeloid neoplasia (MN). We evaluated whether a simple flow cytometry immunophenotyping (FCIP) assay could differentiate the risk of development of MN in patients with CCUS.

**Methods:**

Bone marrow aspirates were assessed by FCIP panel in a cohort of 80 patients identified as having CCUS based on next-generation sequencing or cytogenetics from March 2015 to May 2020, with available samples. Flow cytometric assay included CD13/HLA-DR expression pattern on CD34-positive myeloblasts; CD13/CD16 pattern on maturing granulocytic precursors; and aberrant expression of CD2, CD7, or CD56 on CD34-positive myeloblasts. Relevant demographic, comorbidity, and clinical and laboratory data, including the type and extent of genetic abnormalities, were extracted from the electronic health record.

**Results:**

In total, 17 (21%) patients with CCUS developed MN over the follow-up period (median survival follow-up, 28 months [95% confidence interval, 19-31]). Flow cytometry immunophenotyping abnormalities, including the aberrant pattern of CD13/HLA-DR expression, as detected at the time of the diagnosis of CCUS, were significantly associated with risk of developing MN (hazard ratio, 2.97; *P* = .006). Additional FCIP parameters associated with the development of MN included abnormal expression of CD7 on myeloblasts and the presence vs absence of any FCIP abnormality.

**Conclusions:**

A simple FCIP approach that includes assessment of CD13/HLA-DR pattern on CD34-positive myeloblasts can be useful in identifying patients with CCUS at higher risk of developing MN.

KEY POINTS

- Flow cytometry immunophenotyping can identify patients with clonal cytopenia of undetermined significance (CCUS) at risk of progression to chronic myeloid neoplasm.
- CD13/HLA-DR expression on myeloblasts is a good predictive marker of CCUS progression.
- Simple qualitative flow cytometry panels still have a place in an increasingly quantitative world of flow cytometry.

## INTRODUCTION

Chronic myeloid neoplasias (MNs) are diseases of hematopoiesis arising from the accumulation of genetic abnormalities in hematopoietic stem cells. They include myeloproliferative neoplasms (MPNs), myelodysplastic syndrome (MDS), overlap disorders (MDS/MPN), and several other rarer entities.^1^ Diagnostic criteria for MNs are based on the integration of clinical features, morphologic assessment of the peripheral blood and bone marrow, and cytogenetic and molecular findings.

Chronic MNs are usually slowly developing diseases, but several precursor conditions are diagnosed clinically in the absence of definitive morphologic and genetic features of MN: (1) clonal hematopoiesis of indeterminate potential (CHIP), characterized by the presence of pathologic mutations with low allele frequency in the absence of other clinical, morphologic, and genetic abnormalities^2^; (2) idiopathic cytopenias of undetermined significance, a diagnosis of exclusion rendered when no other explanation for cytopenias is seen and no cytogenetic (structural variation or somatic copy number alterations) or molecular abnormalities are present^3^; and (3) clonal cytopenias of undetermined significance (CCUS), characterized by both cytopenias and the presence of pathologic mutations/somatic copy number alterations but still with morphologic and genetic findings insufficient for the definitive diagnosis of MN.^2,4-6^

Identification of phenotypic abnormalities on maturing myeloid cells by flow cytometric immunophenotyping (FCIP) can be useful in the diagnosis of chronic MNs^7,8^ and possibly in predicting the development of MNs from precursor lesions.^9^ Flow cytometric abnormalities are not considered definitively diagnostic, however, for chronic MNs in the absence of morphologic or cytogenetic abnormalities.^1,6^

It has previously been demonstrated that normal CD34-positive myeloblasts (immature myeloid precursors) show heterogeneous expression of CD13/HLA-DR and that this heterogeneity is lost in a significant number of cases with chronic MN.^10^ The sensitivity and specificity of the CD13/HLA-DR expression pattern can be further increased by evaluation of (1) aberrant expression of CD2, CD7, and CD56 on myeloblasts; (2) a CD13/CD16 pattern of maturation in granulocytic lineage cells; (3) the ratio of CD45 expression between myeloblasts and lymphocytes; and (4) the ratio of side scatter (SSC) between granulocytes and lymphocytes.

In this study, we evaluated the clinical value of this approach based on the retrospective review of flow cytometry findings from bone marrow evaluation in 80 patients with a diagnosis of CCUS.

## MATERIALS AND METHODS

This retrospective registry study conducted through our hematopathology group was approved by our institutional review board.

### Patients

In total, 80 patients with a diagnosis of CCUS from 2015 to 2020 were included in the study. Initial patient selection was based on the availability of the FCIP results. Relevant demographic, clinical, and laboratory data were extracted from the electronic health record. From a larger cohort of patients, the CCUS cohort presented here was selected based on the clinical findings (cytopenia), morphologic bone marrow assessment at the time of the FCIP study (lack of diagnostic features of MN), and genetic studies (presence of a clone by molecular or cytogenetic study). The diagnosis of CCUS was made based on the International Consensus Group criteria.^6^ The patients were followed up at our institution clinically and with subsequent bone marrow assessment. All but 2 of the patients who developed MN have undergone a second bone marrow examination.

### Pathology Assessment

All bone marrow samples were assessed for the presence of MN according to the World Health Organization (WHO) 2016 classification. Additional immunohistochemical, cytogenetic, and molecular studies were performed at the discretion of the original reviewing pathologist. There were 7 cases with subsequently detected mutation in the *SF3B1* gene that were included in this cohort because of (1) the absence of morphologic dysplasia in at least 10% of the erythroid cells or (2) the absence of ring sideroblasts.

**Figure 1:**
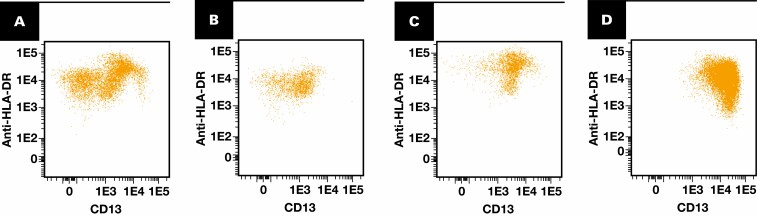
Examples of CD13/HLA-DR patterns on CD34-positive myeloblasts. **A**, Normal pattern. **B**, **C**, Atypical examples still show a certain degree of heterogeneity of CD13/HLA-DR expression but with loss of the typical pattern seen in normal bone marrow. **D**, Abnormal pattern.

Flow cytometric immunophenotyping was performed on initial bone marrow aspirate specimens as previously described.^10^ Bone marrow aspirates were processed using a lyse-wash-stain procedure and stained in 2 8-color tubes; antibodies and clones are listed in Supplemental Table 1 (all supplemental materials can be found at *American Journal of Clinical Pathology* online). All antibodies were purchased from BD Biosciences. A total of 500,000 events were collected per tube using BD FACSCanto II instruments. The data were analyzed using Kaluza software (Beckman-Coulter). CD34-positive CD45dim myeloblasts were evaluated for aberrant expression of CD2, CD7, and CD56 (at cutoffs 10%, 30%, and 10%, respectively; these cutoffs were established previously by adding 2 standard deviations to the mean of the expression in a validation cohort of samples from individuals with no MNs). In addition, CD34-positive myeloblasts were evaluated for the aberrant expression pattern of CD13/HLA-DR, as previously described.^10^ Briefly, CD34-positive myeloblasts from normal healthy donors show a characteristic pattern of CD13/HLA-DR expression, with 3 readily recognizable clusters Figure 1A. Patients with myeloid neoplasms often lose this heterogeneity and instead show a single prominent abnormal cluster Figure 1D. There are cases in which the visual pattern of expression falls between “normal” and “abnormal,” and these were deemed atypical Figures 1B and 1C. A similar approach (normal, atypical, abnormal) was taken for evaluating granulocyte maturation in the CD13/CD16 plot.^11^ Additional parameters assessed included percentage of CD19-positive hematogones (out of total events), the ratio of CD45 mean fluorescence intensity between lymphocytes and CD34-positive myeloblasts, and the ratio of SSC between granulocytes and lymphocytes. For the current study, all dot plots were re-reviewed independently by 2 board-certified hematopathologists (D.J. and P.L.N.) for confirmation of interpretation and for consensus reconciliation when there were discrepancies with the original interpretation. This review was done blinded to the patients’ final diagnosis.

Cytogenetic testing for structural rearrangements and copy number abnormalities in bone marrow aspirates was performed by conventional chromosome analysis and fluorescence in situ hybridization (FISH) analysis at the hematopathologist’s discretion. Conventional chromosome analysis was performed according to standard unstimulated bone marrow culturing and GTL-banded metaphase preparation methods with trypsin and Leishman staining. Slide preparations were digitally scanned, and metaphase spreads were karyotyped by a technologist using CytoVision software (Leica). Interphase FISH analysis was performed on fresh bone marrow aspirate specimens. Slides were prepared from a fixed-cell suspension and pretreated using traditional cytogenetic methods. Probes were hybridized to specific segments of DNA within the cells on the slide. Recurrent abnormalities in MDS were targeted for FISH analysis, including inv(3)/t(3;3), −5/5q−, −7/7q−, +8, KMT2A rearrangements, −17/17p−, and 20q−/ider(20q). We used 4′,6-diamidino-2-phenylindole dihydrochloride to stain all nuclei. Interphase FISH analysis was performed by 2 technologists using CytoVision software.

Molecular testing for MN-associated mutations was performed using the OncoHeme next-generation sequencing (NGS) panel, which interrogates 35 genes (42 genes starting in November 2018) recurrently mutated in myeloid neoplasms, as previously described.^12^

### Statistical Considerations

Characteristics of the patients with CCUS included in these analyses were summarized using graphical and descriptive statistics. Estimated median survival and corresponding CIs were derived based on Kaplan-Meier methods. Univariate and multivariable Cox regression models were used to evaluate the influence of the various measures as well as the flow markers in relation to MN-free survival and OS. Further, we evaluated the cumulative incidence of MN with a competing risk of death to account for any patients who died before developing MN. All analyses were performed using the R, version 3.6.2 for Windows (R Foundation for Statistical Computing).

## RESULTS

The demographic, clinical, and laboratory features of our analysis cohort are shown in Table 1. Abnormal cytogenetics (non–MDS-defining, per the 2017 WHO classification) were detected in 17 cases and mutations in MN-associated neoplasm by NGS panel in 63 cases. The most common cytogenetic abnormalities were trisomy 8, deletion 20q, and loss of 1 Y chromosome in more than 50% of metaphases (6, 6, and 3 patients, respectively). The most common mutations were found in the *TET2, SRSF2, ASXL1,* and *U2AF1* genes (20, 16, 15, and 12 patients, respectively). The 10 most common genes mutated in this cohort are shown in Table 2; the complete list of mutated genes is shown in Supplemental Table 3. Multiple mutations were present in 35 patients (range, 2-5 mutations; overall mean, 1.5 mutations; median, 1 mutation). The median survival follow-up for these patients was 27.7 months (95% CI, 19.1-30.9). In this cohort of 80 patients with CCUS, 17 (21%) MN events were reported during follow-up (11 MDS; 3 chronic myeloid neoplasm, unspecified; 2 chronic myelomonocytic leukemia; and 1 acute myeloid leukemia) and 21 deaths were reported (7 of which had an MN event reported before death).

**Table 1:** Clinical and Laboratory Characteristics of Patients With Clonal Cytopenia of Undetermined Significance

Characteristic or Marker	Patients With CCUS (n = 80)
Age, median (range), y	72 (19-92)
Sex, No. (%)	
F	21 (26)
M	59 (74)
Splenomegaly, No. (%)	
No	66 (83)
Yes	14 (18)
Prior chemotherapy or RT, No. (%)	
No	70 (88)
Yes	10 (13)
ANC, median (range), ×10^9^/L	2.0 (0-40)
ALC, median (range), ×10^9^/L	1.26 (0.16-40)
WBC, median (range), ×10^9^/L	4.0 (0.3-23)
Hemoglobin, median (range), g/dL	10.5 (6.8-14.9)
Platelet count, median (range), ×10^9^/L	126 (7-595)
High LDH (>222 U/L), No. (%)	
No	23 (50)
Yes	23 (50)
High ferritin (>336 µg/L), No. (%)	
No	31 (39)
Yes	21 (26)
Loss of Y chromosome, No. (%)	
No	76 (95)
Yes	4 (5)
del(20)(q11.q13.1), No. (%)	
No	77 (96)
Yes	3 (4)
Loss of X chromosome, No. (%)	
No	78 (97)
Yes	2 (3)

ALC, absolute lymphocyte count; ANC, absolute neutrophil count; CCUS, clonal cytopenia of undetermined significance; LDH, lactate dehydrogenase; RT, radiation therapy.

**Table 2:** The 10 Most Common Genes Mutated in the Clonal Cytopenia of Undetermined Significance Cohort

Mutation	VAF Range, %	Frequency of Mutation in Patients With CCUS, No. (%) (n = 80)
*TET2*	8-77	20 (25.0)
*SRSF2*	14-52	16 (20.0)
*ASXL1*	9-46	15 (18.75)
*U2AF1*	9-43	12 (15.0)
*SF3B1*	6-45	7 (8.75)
*DNMT3a*	7-43	6 (7.5)
*ZRSR2*	57-88	6 (7.5)
*IDH1*	12-44	5 (6.25)
*TP53*	13-48	5 (6.25)
*RUNX1*	13-50	5 (6.25)

CCUS, clonal cytopenia of undetermined significance; VAF, variant allele frequency.

**Figure 2:**
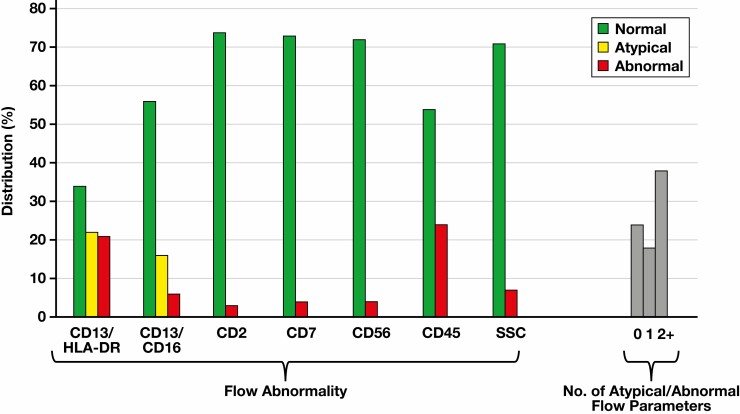
Distribution of flow cytometry immunophenotyping abnormalities seen in patients with clonal cytopenia of undetermined significance.

Of 80 patients with CCUS, the expression pattern of CD13/HLA-DR on CD34-positive myeloblasts was normal, atypical, or abnormal in 34 (42.5%), 22 (27.5%), and 21 (26%) patients, respectively Figure 2. Three patients (4%) did not fulfill the criteria for CD13/HLA-DR evaluation (minimum of 500 CD34-positive myeloblasts collected). The expression pattern of CD13/CD16 on maturing granulocytes was normal, atypical, or abnormal in 56 (70%), 16 (20%), and 6 (7.5%) patients, respectively. Two patients (2.5%) did not fulfill the criteria for CD13/CD16 evaluation (minimum of 30,000 maturing granulocytes collected). Abnormal CD45 expression was observed in 24 patients, whereas abnormal CD7 expression on myeloid myeloblasts was identified in just 4 patients. The distribution of the majority of other FCIP parameters was skewed, with most patients showing normal pattern/expression. The absence of any FCIP abnormality was seen in 24 (30%) patients, while more than 1 abnormality was detected in 38 (47.5%) patients. There was a high correlation between original interpretation of CD13/HLA-DR and CD13/CD16 expression and the subsequent expert review (weighted Cohen κ coefficient, 0.657 and 0.815, respectively) (Supplemental Tables 2A and 2B).

It has been shown in the past that immunophenotypic abnormalities are correlated with both morphologic and genetic abnormalitites.^7,8^ In this cohort, there were 4 mutations, the presence of which was associated with flow cytometric abnormalities: *ASXL1, U2AF1, RUNX1,* and *BCOR.* Specifically, an abnormal CD13/HLA-DR pattern was associated with the presence of *ASXL1, U2AF1,* and *BCOR* mutations (33% vs 12.5%, *P* = .048; 33% vs 7%, *P* = .007; 33% vs 7%, *P* = .007, respectively) (see Supplemental Table 3). As expected, CD13/HLA-DR abnormality was correlated with the total number of mutations present; a Poisson model for the number of mutations showed that there is a significantly higher number of mutations in those with abnormal CD13/HLA-DR expression on flow cytometry than those without (incidence rate ratio estimate of 1.49, *P* = .0375). We also assessed the correlation of CD13/HLA-DR abnormality with the blast count. Although the blast range in the CCUS population is by definition limited, we observed a positive correlation between the blast percentage and the presence of an CD13/HLA-DR abnormality (Supplemental Table 4).

**Figure 3:**
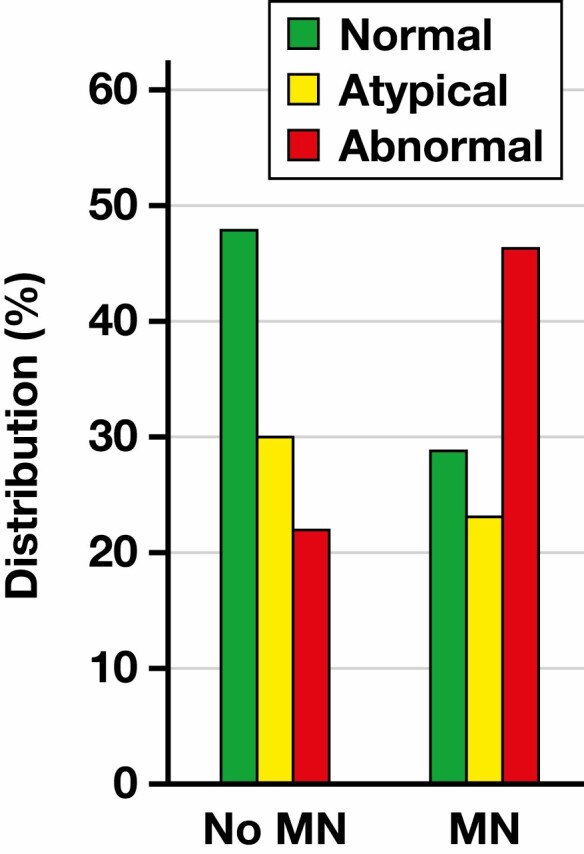
Distribution of CD13/HLA-DR patterns in patients with clonal cytopenia of undetermined significance stratified based on their subsequent progression to myeloid neoplasia (MN).

Across all patients in this CCUS cohort, the median time to development of an MN or death (ie, MN-free survival) was 24.6 months (95% CI, 18.5 to not yet reached). Of 17 patients who developed MN, 5 (29%) had a normal, 4 (24%) had an atypical, and 8 (47%) had an abnormal pattern of CD13/HLA-DR expression. Conversely, in the group of patients who did not develop MN within the follow-up period (n = 60, excluding 3 patients with insufficient blasts), 29 (48%) patients had a normal, 18 (30%) had an atypical, and 13 (22%) had an abnormal pattern of CD13/HLA-DR expression Figure 3. We found that patients with abnormal expression patterns of CD13/HLA-DR had a higher probability or risk of developing MN or death vs those with normal or atypical patterns in the univariate setting (hazard ratio [HR], 2.85 [95% CI, 1.36-5.95]; *P* = .005) Table 3. The significance of abnormal CD13/HLA-DR expression was retained in a multivariable Cox regression model when adjusting for age, whether patients had 2 or more comorbidities (including splenomegaly), and prior exposure to radiation therapy (HR, 2.97 [95% CI, 1.37-6.46]; *P* = .006) Table 4. Similarly, and although abnormal CD7 expression was far less prevalent at the time of CCUS diagnosis in these patients, it was significantly associated with greater risk of MN and death in these patients, even adjusting for age, multiple comorbidities (≥2 vs 0-1) and prior radiation therapy (HR, 4.60 [95% CI, 1.19-17.77]; *P* = .027). Having at least 1 FCIP abnormality was also associated with a greater risk of MN and death (HR, = 2.56 [95% CI, 1.20-5.46]; *P* = .015).

**Table 3:** Cox Regression Univariate Model Results for Flow Markers^a^

Flow Marker	No. With Abnormal Expression	HR (95% CI)	*P* Value
CD13/HLA-DR	21	2.85 (1.36-5.95)	.005
CD7	4	3.49 (1.04-11.71)	.044
CD2	3	0.72 (0.10-5.31)	.75
CD13/CD16	6	1.16 (0.28-4.90)	.84
CD45	24	1.07 (0.50-2.30)	.87
CD56	4	0.49 (0.07-3.58)	.48
SSC	7	1.16 (0.35-3.85)	.81
CD13/HLA-DR, CD13/CD16, or CD7 (vs not any)	26	2.46 (1.19-5.12)	.016

CI, confidence interval; HR, hazard ratio; MN, myeloid neoplasia.

^a^Each row in this table reflects a separate univariate Cox regression model, where the results associated with the marker are presented for risk of MN or death. CD13/CD16, CD2, CD7, CD56, and SSC should be interpreted with caution because of the small number of patients (<10) with abnormal expression of those single markers.

**Table 4:** Cox Regression Model Results for Flow Markers When Adjusting for Age, Having 2 or More Comorbidities, and Prior Radiation Therapy Exposure^a^

		Adjusting for All Covariates		Adjusting Just for Comorbidities	
Flow Marker	No. With Abnormal Expression	HR (95% CI)	*P* Value	HR (95% CI)	*P* Value
CD13/HLA-DR	21	2.97 (1.37-6.46)	.006	2.68 (1.29-5.61)	.009
CD7	4	4.60 (1.19-17.77)	.027	4.70 (1.35-16.39)	.015
CD2	3	0.72 (0.10-5.43)	.75	0.65 (0.09-4.80)	.67
CD13/CD16	6	1.11 (0.25-4.85)	.89	1.03 (0.24-4.36)	.97
CD45	24	0.94 (0.42-2.10)	.88	0.87 (0.40-1.92)	.73
CD56	4	0.36 (0.05-2.75)	.32	0.32 (0.04-2.40)	.27
SSC	7	0.90 (0.26-3.14)	.87	1.06 (0.32-3.52)	.93
CD13/HLA-DR, CD13/CD16, or CD7 (vs not any)	26	2.56 (1.20-5.46)	.015	2.37 (1.14-4.93)	.021

CI, confidence interval; HR, hazard ratio; MN, myeloid neoplasia.

^a^Each row in this table reflects a separate multivariable Cox regression model, where the results associated with the marker are presented for risk of MN or death when adjusting for all covariates of interest as well as for having ≥2 comorbidities, which was the most influential of the 3 on risk of MN. CD13/CD16, CD2, CD7, CD56, and SSC should be interpreted with caution because of the small number of patients (<10) with abnormal expression of those single markers.

**Figure 4:**
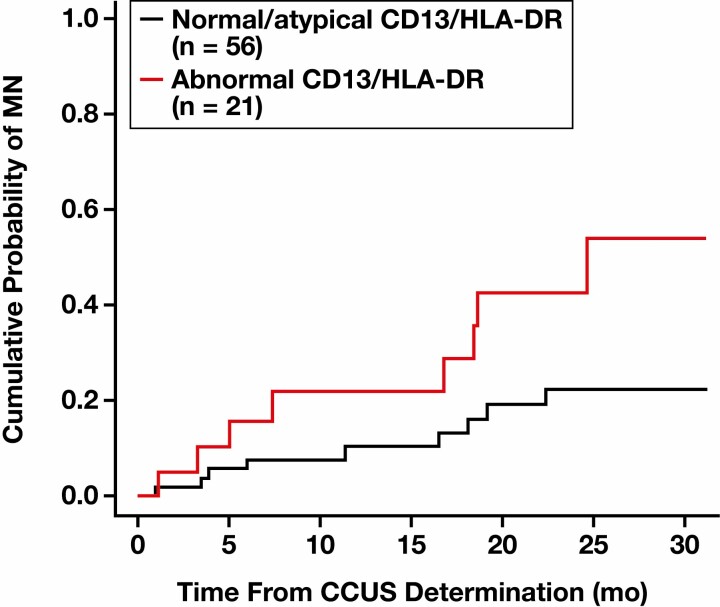
Cumulative probability of developing myeloid neoplasia (MN) based on the expression pattern of CD13/HLA-DR. CCUS, clonal cytopenia of undetermined significance.

We also evaluated cumulative incidence of MN in this CCUS cohort, where death before MN diagnosis was treated as a competing risk as opposed to being considered part of the defined event of interest. In this setting, abnormal expression of CD13/HLA-DR had a significant influence on the probability of developing MN vs those with normal or atypical expression (HR, 2.67 [95% CI, 1.06-6.71]; *P* = .037]) Figure 4 and Table 5. This influence was not observed when looking at death without MN (HR, 1.71 [95% CI, 0.58-5.06]; *P* = .33).

**Table 5:** Probability of Developing Myeloid Neoplasia

	Normal/Atypical CD13/HLA-DR, %	Abnormal CD13/HLA-DR, %
MN		
1 y	10.3	21.7
2 y	22.3	42.4
Death without MN		
1 y	8.4	10.9
2 y	20.4	23.2

MN, myeloid neoplasia.

## DISCUSSION

Despite its more frequent use today, FCIP remains an adjunct method in the diagnosis of MN. Large immunophenotypic variations are seen in normal/reactive conditions, causing the relatively low sensitivity and specificity of any single abnormal parameter. As a result, most FCIP approaches incorporate scoring systems to integrate aberrancies seen in multiple parameters. In any scoring system, the more abnormal parameters are detected, the higher the likelihood of an MN.^13-15^ This finding is similar to the correlation between the number and variant allele frequency of detected mutations and the likelihood of developing MN in patients with CHIP and CCUS.^16^

There is great variability between laboratories in approaches, antibodies, and gating strategy in evaluating myeloid phenotypic aberrancies. Large FCIP panels, as suggested by the European LeukemiaNet Working Group and EuroFlow Consortium,^7,17^ offer a more comprehensive picture and outperform limited panels.^18-20^ Clinical use of large FCIP panels in the diagnosis of MNs, however, has 2 important obstacles. First, the large panels with 20 or more antibodies are expensive to implement, and the reimbursement for testing varies, particularly in the United States. Second, these assays are generally complex from both technical and interpretative aspects; as a result, they are available only in large academic centers. In contrast, limited panels are readily available and can easily be implemented in small laboratories with limited resources and more focused expertise.

It has been shown that abnormal expression of CD13/HLA-DR on CD34-positive myeloblasts is a reproducible finding in a large proportion of MNs.^10^ Here, for the first time, we show that the abnormal expression pattern may precede the clinical diagnosis of an MN. In this study, we evaluated CD13/HLA-DR expression in a cohort of patients with CCUS. We found a positive correlation between the presence of an abnormal CD13/HLA-DR pattern and the likelihood of developing MN. As expected, the presence of an abnormal CD13/HLA-DR pattern was also correlated with the morphologic blast counts and the total number of mutations detected. Although some of the specific mutations also showed a correlation with CD13/HLA-DR abnormalities, it is unclear how this correlation fits into the previously recognized categories of patients with CCUS^16,21^ because the number of patients in specific co-mutational groups was too low for statistical analysis. Patients with CCUS are at high risk of developing MN^21^ and are closely followed, both clinically and with repeated bone marrow assessment. This requirement presents a potential burden on both the patient and the health care system and implies a need for a more precise risk assessment of MN development in these patients. Our findings show that a simple FCIP assessment of myeloblasts, using a limited antibody panel, could be a useful tool that results in better prediction of MN development in patients with CCUS. Importantly, the interpretation of CD13/HLA-DR dot plots has been consistent among 20 hematopathologists: rereview by 2 hematopathologists in the current study (D.J. and P.L.N.) showed a high level of concordance with the original interpretation. Abnormal CD7 expression on CD34-positive myeloblasts was rarely detected (4/80 patients [5%]), but its presence was correlated with a higher likelihood of developing MN. This finding is consistent with previously described risk of developing therapy-related MN after autologous stem cell transplant.^22^ Interestingly, only 4 of the patients with CCUS had aberrant co-expression of CD56 on myeloblasts; none of these patients developed MN during the follow-up period.

Over the past several years, there has been a trend in turning FCIP from a qualitative (interpretative) to a quantitative (exact) analytic tool. Quantitative assessment offers benefits that include greater precision and a reduced requirement for interpretative training. Additionally, a quantitative approach can more readily adopt the use of artificial intelligence– and deep neural network–based tools, as a recent study showed.^23^ Such an approach requires extensive standardization of reagents, staining, and acquisition procedures, however, which is difficult to implement in clinical practice. Therefore, qualitative assessment of FCIP dot plots, as used in this study, is likely to persist for the foreseeable future.

This study has its limitations. It is a single-institution retrospective study with a relatively small number of cases. Although unlikely, there is also a possibility that the subsequent diagnosis of MN in patients with CCUS was influenced by the potential presence of FCIP abnormalities in the original specimen. The FCIP study was not repeated in most follow-up specimens to assess for persistence of abnormalities.

We present evidence that a simple FCIP panel that includes assessment of CD13/HLA-DR expression on CD34-positive myeloblasts could be a useful predictor of MN development in patients with CCUS. Future work is needed to incorporate this finding into an actionable, predictive scoring system for patients with CCUS.

## Supplementary Material

aqac083_suppl_supplementary_Material
