# Supplementary material for: Abnormal CD13/HLA-DR Expression Pattern on Myeloblasts Predicts Development of Myeloid Neoplasia in Patients With Clonal Cytopenia of Undetermined Significance
Source: Am J Clin Pathol. 2022 Aug 8;158(4):530–6. doi: 10.1093/ajcp/aqac083 (PMC9535519; doi:10.1093/ajcp/aqac083)
Supplement: aqac083_suppl_supplementary_Material [file aqac083_suppl_supplementary_material.docx]

**Supplemental Table 1: Antibodies (clones) and fluorochromes used for flow cytometry immunophenotyping**

|  | **FITC** | **PE** | **PercP Cy5.5** | **Pe-Cy7** | **APC** | **APC-H7/Fire** | **V450** | **V500** |
| --- | --- | --- | --- | --- | --- | --- | --- | --- |
| **Tube 1** | **HLA-DR**  (L243) | **CD13**  (L138) | **CD34**  (8G12) | **CD33**  (P67.6) | **CD117**  (104D2) | **CD16**  (3G8) | **CD15**  (MMA) | **CD45**  (HI30) |
| **Tube 2** | **CD2**  (S5.2) | **CD7**  (M-T701) | **CD34**  (8G12) | **CD56**  (NCAM16.2) | **CD36**  (CB38) | **CD64**  (10.1) | **CD38**  (HB7) | **CD45**  (HI30) |

**Supplemental Table 2A: Agreement of interpretative assessment of CD13/HLA-DR pattern on CD34^+^ myeloblasts.**

|  | | **CD13/HLA-DR original interpretation (74 available)** | | |
| --- | --- | --- | --- | --- |
|  |  | **Normal** | **Atypical** | **Abnormal** |
| **CD13/HLA-DR review** | **Normal** | 39 | 5 | 0 |
|  | **Atypical** | 9 | 7 | 0 |
|  | **Abnormal** | 2 | 1 | 11 |

**Supplemental Table 2B: Agreement of interpretative assessment of CD13/CD16 pattern on maturing granulocytic cells.**

|  | | **CD13/CD16 original interpretation (78 available)** | | |
| --- | --- | --- | --- | --- |
|  |  | **Normal** | **Atypical** | **Abnormal** |
| **CD13/CD16 review** | **Normal** | 52 | 2 | 0 |
|  | **Atypical** | 2 | 15 | 0 |
|  | **Abnormal** | 1 | 2 | 4 |

**Supplemental Table 3: Distribution of NGS-detected mutations their association with flow cytometric abnormalities**

| **Mutation** | **VAF range (%)** | **Frequency of mutation in CCUS pts**  **N=80** | **Abnormal CD13/HLA-DR** | | | | **Abnormal CD45** | | | | **Abnormal CD13/HLA-DR, CD13/CD16, and/or CD7 (vs. not any)** | | | |
| --- | --- | --- | --- | --- | --- | --- | --- | --- | --- | --- | --- | --- | --- | --- |
|  |  |  | **No**  **(n=56)** | **Yes**  **(n=21)** | **p-value** | **No**  **(n=54)** | | **Yes**  **(n=24)** | **p-value** | **No**  **(n=51)** | | **Yes**  **(n=26)** | **p-value** |  |
| TET2 | 8-77 | 20 (25.0%) | 16 | 4 | 0.59 | 12 | | 8 | 0.40 | 14 | | 6 | 0.79 |  |
| SRSF2 | 14-52 | 16 (20.0%) | 13 | 2 | 0.22 | 7 | | 8 | 0.059 | 11 | | 4 | 0.76 |  |
| **ASXL1** | 9-46 | 15 (18.75%) | 7 | 7 | **0.048** | 9 | | 5 | 0.75 | 4 | | 10 | **0.003** |  |
| **U2AF1** | 9-43 | 12 (15.0%) | 4 | 7 | **0.007** | 8 | | 4 | 0.99 | 3 | | 8 | **0.006** |  |
| SF3b1 | 6-45 | 7 (8.75%) | 7 | 0 | 0.18 | 7 | | 0 | 0.093 | 7 | | 0 | 0.088 |  |
| DNMT3a | 7-43 | 6 (7.5%) | 3 | 3 | 0.34 | 5 | | 1 | 0.66 | 3 | | 3 | 0.40 |  |
| ZRSR2 | 57-88 | 6 (7.5%) | 3 | 3 | 0.34 | 3 | | 3 | 0.36 | 3 | | 3 | 0.40 |  |
| IDH1 | 12-44 | 5 (6.25%) | 3 | 1 | 0.99 | 3 | | 1 | 0.99 | 3 | | 1 | 0.99 |  |
| TP53 | 13-48 | 5 (6.25%) | 5 | 1 | 0.99 | 5 | | 1 | 0.66 | 5 | | 1 | 0.66 |  |
| **RUNX1** | 13-50 | 5 (6.25%) | 2 | 3 | 0.12 | 1 | | 4 | **0.029** | 2 | | 3 | 0.33 |  |
| **BCOR** | 19-38 | 4 (5.0%) | 0 | 4 | **0.004** | 0 | | 4 | **0.007** | 0 | | 4 | **0.011** |  |
| IDH2 | 31-47 | 4 (5.0%) | 3 | 1 | 0.99 | 2 | | 2 | 0.58 | 2 | | 2 | 0.60 |  |
| CB1 | 13-44 | 2 (2.5%) |  |  |  |  | |  |  |  | |  |  |  |
| BRAF | NA | 1 (1.25%) |  |  |  |  | |  |  |  | |  |  |  |
| NPM1 | 32 | 1 (1.25%) |  |  |  |  | |  |  |  | |  |  |  |
| NRAS | 34 | 1 (1.25%) |  |  |  |  | |  |  |  | |  |  |  |
| SETBP1 | 50 | 1 (1.25%) |  |  |  |  | |  |  |  | |  |  |  |
| ETV6 | 45 | 1 (1.25%) |  |  |  |  | |  |  |  | |  |  |  |
| EZH2 | 50 | 1 (1.25%) |  |  |  |  | |  |  |  | |  |  |  |
| JAK2 | 31 | 1 (1.25%) |  |  |  |  | |  |  |  | |  |  |  |
| TERT | NA | 1 (1.25%) |  |  |  |  | |  |  |  | |  |  |  |
| GATA2 | 48 | 1 (1.25%) |  |  |  |  | |  |  |  | |  |  |  |

**Supplemental Table 4: Correlation between flow cytometric abnormalities and morphologic blast count**

| **Flow marker** | **% morphologic blasts** | | | | | | | | | |
| --- | --- | --- | --- | --- | --- | --- | --- | --- | --- | --- |
|  | ***Median (range)*** | ***N in flow group*** | ***Missing blasts data*** | ***0-2%*** | ***3-5%*** | ***p-value*** | ***0-1%*** | ***2-3%*** | ***4-5%*** | ***p-value*** |
| ***CD13/HLA-DR abnormal?***  No  Yes | 1 (1 – 5)  3 (0 – 5) | 56  21 | 17  4 | 34  5 | 5 (13%)  12 (71%) | **<0.0001** | 29  1 | 9 (23%)  8 (47%) | 1 (3%)  8 (47%) | **<0.0001** |
| ***CD45 abnormal?***  No  Yes | 1 (0 – 5)  2 (0 – 5) | 54  24 | 15  7 | 30  9 | 9 (23%)  8 (47%) | 0.11 | 25  5 | 10 (26%)  7 (41%) | 4 (10%)  5 (29%) | **0.042** |
| ***CD13/HLA-DR, CD13/CD16, and/or CD7 abnormal?***  No  Yes | 1 (0 – 5)  3 (0 – 5) | 51  26 | 15  6 | 31  8 | 5 (14%)  12 (60%) | **0.0006** | 26  4 | 9 (25%)  8 (40%) | 1 (3%)  8 (40%) | **<0.0001** |
